# Orofacial Migraine—A Narrative Review

**DOI:** 10.3390/jcm13195745

**Published:** 2024-09-26

**Authors:** Rafael Benoliel, Arne May

**Affiliations:** 1 Department of Diagnostic Sciences, Rutgers School of Dental Medicine, Rutgers University, Newark, NJ 07103, USA; 2 Department of Systems Neuroscience, University Medical Center Hamburg-Eppendorf, 20251 Hamburg, Germany; a.may@uke.uni-hamburg.de

**Keywords:** orofacial migraine, migraine, orofacial pain, facial pain, sinus migraine, sinusitis

## Abstract

The diagnosis of migraine is based on clear criteria outlined in the International Classification of Headache Disorders version 3 (ICHD-3). Notably, the criteria in ICHD-3 omit the location of the migraine. There are increasing reports of migraine in the facial region. Facial presentations of migraine are not easy to diagnose as they appear in the lower two-thirds of the face, often in the maxillary sinus region, around the ear, the upper/lower jaws, and the teeth. Additionally, a similar but distinct entity, neurovascular orofacial pain, has been established. The symptomatology of facial presentations of these headaches often resembles sinusitis and dental pathology. We will review these presentations, their diagnosis, and possible pathophysiology.

## 1. Introduction

Migraine is a common primary headache with an additional number of rarer related syndromes [1]. The combination of high prevalence, severe pain, and debilitating neurological symptoms increases the social impact of migraine beyond that of other primary headaches. The two most common types of migraine headaches are migraine without aura and migraine with aura, which are often confused in the clinic and in the literature [2].

The diagnosis of migraine is based on clear criteria outlined in ICHD-3. Notably, the criteria in ICHD-3 omit the location of the migraine [1], which is unlike the system used for other primary headaches and attests to the variability in pain location [3]. Of relevance are reports of migraine in the facial region [1,4,5,6]. Similarly, facial pain resembling TACs, particularly cluster headaches [6], has also been reported [4,6,7,8,9,10,11,12,13,14,15,16,17,18,19,20,21], most recently in a large patient cohort [5].

The publication of the recent ICOP [22] bridges the classification gap for various OFPs not included in ICHD-3. Section 5 of the ICOP deals with orofacial presentations of primary headaches. The TAC equivalents are beyond the scope of this review (Table 1). Of relevance to this article is that ICOP clearly classifies orofacial presentations of primary migraine and neurovascular OFPs (Table 1, Table 2, Table 3 and Table 4). 

Facial presentations of primary headaches are not easy to diagnose as they appear in the lower two-thirds of the face, often in the maxillary sinus region, around the ear, the upper/lower jaws, and the teeth [23]. Moreover, the symptomatology of facial presentations of headache often resembles sinusitis and dental pathology [24]. Clearly, these cause significant diagnostic difficulties. Little data are available on the epidemiology of orofacial migraine. Based on the existing literature, we estimate a prevalence of 1% of the population. The pathophysiology is likely based on that of migraine (see below) but needs further study. 

### Data Collection

The English literature was searched via PubMed. Search terms included the following: facial X migraine; oral X migraine; orofacial X migraine; sinus X migraine; lower X migraine; lower-half X migraine; neurovascular X orofacial pain; neurovascular X facial pain. 

## 2. The Relation between Head and Face Pain in Patients

The ICOP is not only a classification of orofacial pain but also a bridge between the intimately related subjects of oral, face, and head pain. On the topic of OFPs resembling presentations of primary headaches, the ICOP underscores that in clinical practice, we often see three types of patients who seem to typify the overlap between headache and orofacial pain. Type 1: This includes headache patients who report additional facial pain during, and usually ipsilateral to, the headache attacks. Facial pain may often occur independent of the co-occurrence of headache. Type 2: This includes headache patients whose headache attacks have stopped and been replaced by facial pain attacks of the same quality, length, and intensity, including the occurrence of the associated symptoms of the former headache. Type 3: This includes headache-naive patients who develop de novo orofacial pain attacks that resemble one of the primary headache types in pain character, duration, and intensity, with or without the associated symptoms of these headache types.

**Table 1:** Orofacial pain resembling presentations of primary headaches.

ICOP Coding	Diagnosis	Subcategory Diagnosis/Time Pattern	Time Pattern
5.1	Orofacial migraine		
5.1.1		Episodic	
5.1.2		Chronic	
5.2	Tension-type orofacial pain		
5.3	Trigeminal autonomic orofacial pain		
5.3.1		Cluster orofacial attacks	
5.3.1.1			Episodic
5.3.1.2			Chronic
5.3.2		Paroxysmal hemifacial pain	
5.3.2.1			Episodic
5.3.2.2			Chronic
5.3.3		Short-lasting unilateral neuralgiform facial pain attacks with cranial autonomic symptoms (SUNFA)	
5.3.3.1			Episodic
5.3.3.2			Chronic
5.3.4		Hemifacial continuous pain with autonomic symptoms	
5.4	Neurovascular orofacial pain		
5.4.1		Short-lasting	
5.4.2		Long-lasting	

**Table 2:** Episodic orofacial migraine (ICOP 5.1.1).

	Criteria	Comment
A.	At least five attacks fulfilling criteria B–D.	Episodic or chronic pain exclusively in the orofacial region, without head pain and with the characteristics and associated features of 1. Migraine described in ICHD-3.
B.	Facial and/or oral pain, without head pain, lasting 4–72 h (untreated or unsuccessfully treated).	Orofacial pain otherwise meeting the criteria for any of the subtypes or subforms below, but accompanied by head pain, should be classified according to ICHD-3 under 1. Migraine.
C.	Pain has at least two of the following four characteristics: 1. Unilateral location; 2. Pulsating quality; 3. Moderate or severe intensity; 4. Aggravation by, or causing avoidance of, routine physical activity (e.g. walking or climbing stairs).	Pain displays the typical characteristics of 1. Migraine headache as defined in ICHD-3. A group of patients with attacks of intraoral pain of varying duration, with atypical migraine-like features, have been described. These may be unrelated to migraine, and are described under Neurovascular orofacial pain.
D.	Pain is accompanied by one or both of the following: 1. Nausea and/or vomiting; 2. Photophobia and phonophobia.	Orofacial migraine with aura has not, to our knowledge, been described and is excluded from the ICOP until better evidence of it accumulates.
E.	Not better accounted for by another ICOP or ICHD-3 diagnosis.	

**Table 3:** Chronic orofacial migraine (ICOP 5.1.2).

	Criteria	Comment
A.	Facial and/or oral pain, without head pain, on ≥15 days/month for >3 months and fulfilling criteria B and C below.	The characterization of frequently recurring OFPs generally requires a pain diary to record information on pain and associated symptoms day-by-day for at least 1 month. The occurrence of the equivalent of medication overuse headache in the facial region has not been reported but drug abuse should be considered in chronic facial pain.
B.	Occurring in a patient who has had at least five attacks fulfilling criteria B–D for 5.1 Episodic orofacial migraine.	
C.	On ≥8 days/month for >3 months, fulfilling either of the following: 1. Criteria C and D for 5.1.1 Episodic orofacial migraine; 2. Believed by the patient to be orofacial migraine at onset and relieved by a triptan or ergot derivative.	Pain displays the typical characteristics of migraine headache as defined in ICHD-3.
D.	Not better accounted for by another ICOP or ICHD-3 diagnosis.	

**Table 4:** Diagnostic criteria for neurovascular orofacial pain (NVOP).

Diagnostic Criteria		Notes and Comments
A	At least five attacks of unilateral intraoral pain of variable duration, without head pain, fulfilling criteria B–D.	Although essentially an intraoral pain, there may be referral and/or radiation to adjacent sites, particularly when pain is severe.
B	Pain has both of the following characteristics: 1. Moderate or severe intensity; 2. Either or both of the following qualities: (a) Toothache-like; (b) Pulsating.	Side shift may occur; although pain is mostly unilateral, bilateral cases are reported in up to a third of cases.
C	Pain is accompanied by at least one of the following: 1. Ipsilateral lacrimation and/or conjunctival injection; 2. Ipsilateral rhinorrhea and/or nasal congestion; 3. Ipsilateral cheek swelling; 4. Photophobia and/or phonophobia; 5. Nausea and/or vomiting.	There are reports of abnormal sensitivity to cold, both interictally and during attacks.
D	Pain is unexplained by any local cause, and clinical and radiographic examinations are normal.	Frequently painful vital teeth will be hypersensitive to cold stimuli.
E	Not better accounted for by another ICOP or ICHD-3 diagnosis.	

### 2.1. Facial Presentations of Migraine: Orofacial Migraine

There have a been several reports on migraine-like pain in the lower two-thirds of the face [4,5,10,12,18,25]. There were no clear and consistent criteria for the diagnosis and different terms were assigned, such as orofacial migraine, lower-half migraine, migraine with isolated facial pain [26], or migraine presenting as isolated facial pain [27].

The phenomenon, still poorly recognized, is not new. In 1963, Woolf [28] wrote “*The sites of migraine headache are notably temporal, supraorbital, frontal, retrobulbar, parietal, auricular, and occipital. However... they may occur as well in the malar region the upper and lower teeth, at the base of the nose, in the median wall of the orbit, in the neck*”. In this light, it is surprising that this presentation has caused diagnostic difficulties. As stated above, careful examination of the ICHD-3 criteria for migraine reveals that the location of pain is unspecified [1]. In the footnotes for migraine, the following appears: “a subset of otherwise typical patients has facial location of pain, which is called ‘facial migraine’ in the literature; there is no evidence that these patients form a separate subgroup of migraine patients”. 

Ours and the experience of others confirms this and further suggests that in addition to an orofacial component in migraine attacks, OFM can be totally isolated from head pain [4,10,12,18,25,27]. Often, these isolated facial pains present with a clinical phenotype that, other than the location, may be diagnosed as a migraine. The unusual location, both in migraine and TACs, often leads to erroneous diagnoses relevant to our discussion, such as oral pathology or sinusitis [4,9,10,15,17,19,20,21,29,30,31,32,33,34,35]. Patients with self-reported sinusitis that is migraine pain will respond well to sumatriptan [36]. Whether due to increased education or interest, the diagnosis of head and face pain has shown signs of improvement [37]. 

There are little data specifically on the features of OFM. However, other than location, the clinical phenotype should be the same as in migraine—otherwise, the diagnosis of OFM cannot be established. The ICOP has defined episodic and chronic OFM (Table 2 and Table 3). 

### 2.2. Epidemiology

A population-based study on migraine patients showed that although OFM was not unusual in migraine (8.9%), *isolated* OFM was exceptionally rare (0.2%) [38]. The study findings are limited as the screening questions studied only migraine sufferers and which ones reported concomitant or additional OFM [38]. Patients with isolated OFM without concomitant or prominent symptoms would have been excluded. A study of 1176 patients with migraine [27] found 58 with isolated OFM. The cohort in that study [27] seems mixed, with both OFM and NVOP patients. Pain location was restricted to intra- or/and extraoral areas; two-thirds of these patients underwent endodontics or multiple extractions and around 45% had CASs.

Onset age in NVOP has traditionally been reported at a significantly higher age (around 40–50 years of age) [4,10] than in typical migraine (onset before the age of 20 years in about half of the cases) [39]. A recent study found that the mean age of a patient cohort was 38.5 ± 19.8 years, but it seems to occur in children as well [40,41]. This is like the pattern seen in typical migraine. 

### 2.3. Clinical Features 

*Location/Severity.* The vast majority of patients report moderate-to-severe unilateral pain [27]. Pain occurs primarily in the midface and may resemble sinusitis [15]. It may also appear in the lower face, over the mandible [5,12,17,27]. In a recent study including 1983 migraine patients, 44 had a facial component (2.3%) [5]. Only one patient had a totally isolated OFM and five had a ‘relocated’ migraine. The rest (n = 38) had ongoing migraine with an orofacial component. The most common facial involvement was reported in the maxilla, often involving dental pain. The mandible was affected in only four cases of facial involvement (9.3%) [5].

*Quality and Temporal Pattern.* Pain is usually throbbing and mimics inflammatory pathologies of the maxillary sinuses and the teeth. One would assume that duration is the same as in migraine, but the variability has not been described. Although migraine is typically episodic, there are case series displaying a chronic course, for example, up to 66% in the Lambru et al. series [27]. This is like that seen in NVOP (see below). 

*Accompanying Phenomena.* Research has shown that patients with isolated OFM report significantly more CASs, including conjunctival injection, tearing, miosis, ptosis, eyelid oedema, nasal congestion, and facial flushing, than other migraine patients (47.8% vs. 7.9%) [27,38]. Also, it is important to emphasize that these facial presentations of headache disorders, with mixed migraine and trigeminal autonomic characteristics, are often misdiagnosed and many times mistreated as dental or otolaryngological problems [12,27,31]. No aura has, at this point, been reported in OFM (type 2 or 3) [5].

*Treatment Response.* Migraine therapy should be effective in OFM, but we are still lacking data.

*Differential diagnosis.* OFM is not a straightforward diagnosis and is often masked by its atypical location and features [6]. OFM may mimic facial forms of CH and a number of atypical facial pains [6]. Confusion with sinusitis [11,15] and dental pain [31] is common. 

## 3. Neurovascular Orofacial Pain

The ICOP defines OFPs resembling presentations of primary neurovascular headache under the following three categories: orofacial migraine, orofacial TACs, and NVOP. The ICOP subdivided NVOP into short- and long-lasting forms, reflecting the phenotypes that have been reported (Table 4) [4,10,42,43]. NVOP shares many migraine signs and symptoms, such as pulsating quality, often accompanied by nausea and/or vomiting, photophobia, and phonophobia. Additionally, NVOP includes signs and symptoms such as toothache and dental sensitivity to cold, while CASs include signs and symptoms such as ipsilateral lacrimation and/or conjunctival injection and ipsilateral rhinorrhea and/or nasal congestion. Further research is needed to define the relationship between NVOP and OFM. 

The clinical data suggest that NVOP includes type 2 and type 3 patients that develop migraine as described above in and the OFPs defined in the ICOP. In some cases, a dental intervention such as tooth extraction may result in the “remapping” [44] of an isolated OFM-type pain at the site of intervention. Additionally, it should be noted that in some of the cases mentioned under Type 1 (OFP during a migraine attack), tooth ache in the ipsilateral area is a common complaint. 

Up until the publication of the ICOP, differentiating between the clinical phenotype of OFM and NVOP was not an easy task. Therefore, in our discussions on clinical features and epidemiology here, and above in the Section 3.4, there is undoubtedly an overlap and the cohorts are not 100% homogenous. Going forwards, the relationship between OFM and NVOP needs further clarification. In the interim, we consider the data presented as possibly representing a mixed cohort of OFM and NVOP. We discuss this below in Section 4.3, Differential Diagnosis.

### 3.1. Epidemiology

The onset of NVOP is around 40–50 years of age (mean 43.4 years), with a female/male ratio approaching 4:1 [4,10,26,27,43]. Time to diagnosis ranged from 1 to 528 months, indicating significant diagnostic difficulties [4,12]. In up to two-thirds of cases, the pain was diagnosed as dental pathology and patients underwent unwarranted dental intervention.

### 3.2. Clinical Features 

Table 4 summarizes the features of NVOP, based on the relevant publications [4,26,27,43]. To date, over 199 cases have been published to establish a demographic and clinical database.

*Location.* Pain is overwhelmingly unilateral (76%). Pain occurs mostly intraorally, involving the teeth and the alveolar process (62%). Mucosal sites are less often affected by pain (32%) [4,12,43]. Pain referral is roughly one-third to each of the following areas: perioral structures (lips, chin, etc.), the periorbital region (usually infraorbital), and the preauricular region. 

*Quality and Temporal Pattern.* Severe, pulsating pain (7-8 on VAS) [12] lasts from minutes to hours and up to 3 days [27,43]. Most cases are characterized by a high frequency of attacks of a short duration (60%), exacerbated by cold food ingestion [43]. NVOP is subdivided into a short- and long-lasting (40%) form. Both are mostly recurrent and chronic in nature (Table 2, Table 3 and Table 4) [27].

*Accompanying Phenomena.* The majority of patients (80%) reported CASs, specifically tearing (10–20%), conjunctival injection (14%), miosis (14%), ptosis (3%), and nasal congestion (7–40%), [4,12,27]. Photo/phonophobia (14%) and nausea (24%) may be present [4,12,27]. Very often, patients report dental hypersensitivity to cold, leading to diagnostic confusion with dental pathology [31,45]. 

### 3.3. Treatment 

Low-dose amitriptyline, propranolol, and anti-convulsant therapy have been successful prophylactics in NVOP patients [4,12,27,31,45]. Triptan as an abortive agent was reported in one study and was effective in all patients [26]. It has been our experience that due to the persistent pattern and the exquisite sensitivity to cold food ingestion, prophylactic pharmacologic treatment is preferrable in NVOP.

### 3.4. Differential Diagnosis

Due to the dental thermal hypersensitivity observed in NVOP, the differential diagnosis will include dental pulpitis and cold stimulus, or “ice-cream” headache. Although initially dental pulpitis may resemble NVOP, careful history and examination will differentiate them. Trial triptan treatment may be considered in order to confirm or refute migraine-like mechanisms in NVOP [26]. Cold-stimulus headache occurs particularly in individuals with a history of migraine and is not associated with dental pathology. Pain is evoked by cold material stimulating the palate and posterior pharyngeal. The evoked facial pain is in the mid-frontal region or around the ears, referred probably by the trigeminal and glossopharyngeal nerves, respectively. No treatment other than sensible caution is needed. PTTN may be confused with NVOP due to the prolonged gingival cold allodynia that so often accompanies PTTN [46]. However, PTTN’s history of trauma, the sensory deficits on examination, and the pain quality and pattern are very different from those of NVOP [10,43,46].

NVOP and OFM show great similarity, particularly their response to prophylactic pharmacologic treatment. In a recent study, we also found that these entities were similar in age, sex distribution, and pain intensity [47]. However, OFM patients presented with more CASs, more migraine symptoms (nausea, photophobia), and significantly more pain-related wakening from sleep [47]. OFM pain was more frequently associated with headache. NVOP patients have, by definition, pain that mimics toothache, which is rare in OFM. 

In summary, the diagnostic features of OFM and NVOP indicate that there are many similarities between the two. However, unique features suggest that OFM and NVOP remain as distinct diagnostic entities, in accordance with the ICOP [22]. This is important for both clinical and research purposes.

## 4. Pathophysiology

Migraine headache reflects an altered brain excitability state that is capable of activating the system in genetically susceptible individuals [48]. The hypothalamus and its functional connections to brainstem nuclei and cortical regions underlie the premonitory phase, whereas the headache phase involves increased sensory processing within peripheral and central TMV pathways. Current data indicate that cortical spreading depolarization and activation of the TMV system and its constituent neuropeptides (resulting in neurogenic inflammation), as well as neuronal and glial ion channels and transporters, contribute to the putative cortical excitatory/inhibitory imbalance that renders those with migraine susceptible to an attack [49]. The various symptoms and neurological disturbances observed during all phases of migraine are complex and wide-ranging. Disturbances of sensory function, sensory affect, and cognitive and autonomic function may be experienced, suggesting the involvement of multiple neural networks [50].

### 4.1. Pathophysiology of Facially Located Neurovascular Pain 

The mechanisms underlying facial pain presentations of neurovascular headache disorders are suspected to involve the mechanisms described above but remain unclear [23]. A common hypothesis implicates innervation patterns of the intracranial and extracranial trigeminal nerve. The dura mater is a pain-sensitive intracranial structure and is primarily innervated by the V1 branch, with little arising from V2 and V3. The intra- and extracranial innervations of the trigeminal nerve communicate the presence of pain. Intracranial fibers exit the skull to innervate the periosteum and extracranial tissues, such as the pericranial muscles [51]. This connection provides a route for how TMV activation of the dura extends to the extracranial counterpart, the V1 dermatome in the face [52]. In the posterior cranium, the dura is innervated by intracranial fibers from V2, V3, and cervical branches [53]. Their activation is more likely to cause posterior head pain. Activation of intracranial V1 would evoke frontal headache. 

Neurogenic inflammation induced by intranasal administration of capsaicin and formalin increased plasma protein extravasation not only in the nasal mucosa but also in the dura mater [54]. Thus, extracranial activation of the trigeminal nerves leads to intracranial activation of their counterparts. Based on the anatomical and functional connection between different branches of the trigeminal system, facial presentations of headache should be more prevalent. Migraine in the lower two-thirds of the face accompanied by systemic and CASs raises mechanistic challenges. The pathophysiology of NVOP may be based on migraine. If so, we need to analyze the possibility that neurogenic inflammation (NI) occurs in the oral and perioral tissues and consider its possible role in the phenotype of NVOP.

### 4.2. Neurogenic Inflammation in Oral Tissues and the Dental Pulp

The dental pulp is innervated by unmyelinated C-fibers, autonomic nerves, myelinated A-δ, and A-β fibers. Also present in the dental pulp and oral mucosa in several species including humans are SP- and CGRP-positive immunoreactive fibers [55]. The levels of two sensory neuropeptides (SP and CGRP) and two endogenous opioids, methionine-enkephalin and β-endorphin, were studied in human dental pulps. After 24 h of an intrusive stimulus associated with discomfort, only SP significantly increased, clarifying the role of neurogenic inflammation in early injury response [56]. The human dental pulp has high expression of CGRP, SP, and VIP, and is higher in permanent than in deciduous teeth [57]. This possibly explains the lack of NVOP reported in children [4,12]. Antidromic electrical nerve stimulation induces neurogenic inflammation (NI) in the dental pulp of dogs and in the dental pulp, lower lip, and oral mucosa of rats [58,59], which reduces after sympathectomy [60]. Possible collateral C-fiber innervation in the same quadrant may explain the symptomatology in adjacent teeth [60,61,62]. This phenomenon may underlie the referral patterns in primary NVOP. The anatomical substrate for neurovascular tooth pain is therefore present.

NI in the TMV system seems to play a central role in the genesis of headache and facial pain; so, the same mechanism could function in the oral mucosa and teeth. 

Activation of 5-HT-1B/1D receptors, through local injection of naratriptan into the ventrolateral PAG, produces selective inhibition of TMV nociceptive afferent input but not facial afferents [63]. It is possible that this finding underlies the sparse reports of successful treatment of orofacial neurovascular counter parts (OFM, NVOP, and TACs). 

### 4.3. Central Mechanisms

The central somatotopy is onion-ring shaped, with the center being the perioral region. However, fibers from the V1 branch project more to the caudal part of the TNC, whereas those from the V2 and V3 branches project more to the rostral part of the TNC [64]. This distribution also provides the anatomical basis of why cervical modulation, e.g., greater occipital nerve block, may be effective in aborting headache disorders, since the V1 dermatome projects to the most caudal part of the TNC and locates directly adjacent to the secondary sensory neuron of the C2/C3 branches in the spinal cord [65,66]. Capsaicin stimulation of the V1 dermatome modulated the pain threshold in the V2, V3, and greater occipital nerve dermatome. Stimulation at the greater occipital nerve was able to change the pain threshold on all three branches of the trigeminal nerve, but with a stronger effect on V1 compared to V2/V3 [67]. Thus, functional interactions between different branches of the trigeminal nerve take place at the pontomedullary level. There is a functional connection between the limbic system and the ophthalmic branch [68,69]; this functional connection explains why such attack-like pain in migraine is predominately in the ophthalmic dermatome [70]. Following this thought, the facial presentations would be a simple “spread” of pontomedullary activation in type 1 and type 2 facial presentations of headache, whereas the isolated facial attacks resembling headaches (type 3) are due to rare functional connections between the limbic system and the maxillary or mandibular brainstem nuclei. Further studies into this subject are needed.

## 5. Conclusions

Although facial presentations of neurovascular-type headaches have been recognized for some time, the lack of internationally accepted inclusion criteria has prevented pooling of the data. This would enhance the establishment of the different clinical phenotypes. 

The ICOP has established clear criteria for the diagnosis of facially located migraine and TACs. There is a need to field test the criteria to refine the classification as we approach the time for a second version. Additionally, treatment response needs to be tested in randomized controlled studies. 

The relation of facially located neurovascular headaches to their typical counterparts needs careful comparison, which will offer some insights into the pathophysiology of facially located pain. 

## Abbreviations

CAS = cranial autonomic symptom; CH = cluster headache; ICOP = Classification of Orofacial Pain, 1st edition; ICHD-3 = International Classification of Headache Disorders version 3; NI = neurogenic inflammation; NVOP = neurovascular orofacial pain; OFM = orofacial migraine; PAG = periaqueductal gray; PTTN = painful traumatic trigeminal neuropathy; TAC = Trigeminal Autonomic Cephalgia; TMV = trigeminovascular; TNC = trigeminal nucleus caudalis; 5-HT-serotonin.
